# Long‐term Outcomes of Intraductal Fully Covered Self‐Expandable Metal Stents for Anastomotic Biliary Strictures After Living Donor Liver Transplantation: Clinical and Economic Evaluation

**DOI:** 10.1002/deo2.70264

**Published:** 2025-12-26

**Authors:** Akane Shimakura, Eisuke Ozawa, Mizuki Kitagawa, Kosuke Takahashi, Masanori Fukushima, Ryu Sasaki, Masafumi Haraguchi, Satoshi Miuma, Akihiko Soyama, Susumu Eguchi, Hisamitsu Miyaaki

**Affiliations:** ^1^ Department of Gastroenterology and Hepatology Nagasaki University Graduate School of Biomedical Sciences Nagasaki Japan; ^2^ Department of Surgery Nagasaki University Graduate School of Biomedical Sciences Nagasaki Japan

**Keywords:** anastomotic biliary stricture, endoscopic retrograde cholangiopancreatography, fully covered self‐expandable metal stent, living donor liver transplantation, plastic stent

## Abstract

**Objectives:**

Anastomotic biliary stricture (ABS) is a common complication following living donor liver transplantation (LDLT). Although plastic stents (PSs) have traditionally been the standard treatment, fully covered self‐expandable metal stents (FCSEMSs) have recently gained attention because of their potential advantages. This study aimed to retrospectively analyze cases of ABS after LDLT that were treated with intraductal FCSEMSs (ID‐FCSEMS) at our institution.

**Methods:**

This study included 46 adult patients who developed anastomotic bile duct stricture following LDLT. Twenty patients underwent ID‐FCSEMS placement, and 22 patients underwent PS placement. The FCSEMSs were scheduled for removal after 16 weeks.

**Results:**

Placement of FCSEMSs was technically successful in all 20 patients. Early complications included cholangitis in five patients, whereas late complications included one case of stent migration and one case of obstructive cholangitis. Clinical success was achieved in 16 patients. Restenosis occurred in two patients. No significant differences were found between the FCSEMS group and the PS group in terms of reimbursement points (as defined by the Japanese medical fee schedule), number of hospitalizations, and total inpatient days.

**Conclusions:**

ID‐FCSEMSs demonstrate a high success rate and favorable long‐term outcomes for ABS after LDLT. Although no significant difference in cost reduction is observed, ID‐FCSEMSs are a safe and effective therapeutic option for ABS.

## Graphical abstract

In this single‐center retrospective study of anastomotic biliary strictures after living donor liver transplantation, intraductal fully covered self‐expandable metal stents (ID‐FCSEMSs) achieved durable stricture resolution with low restenosis. Compared with multiple plastic stents, ID‐FCSEMS were associated with fewer late biliary complications and a similar number of endoscopic sessions. Overall medical reimbursement costs were comparable between groups, supporting ID‐FCSEMS as an effective, safe option without added economic burden.

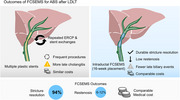

## 1 Introduction

Living donor liver transplantation (LDLT) is an effective treatment for decompensated liver cirrhosis. However, anastomotic biliary stricture (ABS) is one of the most frequent postoperative complications, significantly affecting patient prognosis and quality of life [1, 2]. The incidence of ABS ranges from 10% to 40% in cases of biliary anastomosis following LDLT [3], with most cases occurring within months of transplantation.

Traditionally, endoscopic approaches, particularly staged dilation with plastic stents (PSs) and repeated replacements, have been the standard therapy for ABS [4, 5, 6]. However, this strategy has drawbacks, including prolonged treatment, repeated procedures, and risk of restenosis.

Fully covered self‐expandable metal stents (FCSEMSs) have recently shown favorable outcomes and emerged as a new treatment option for ABS [7, 8]. In particular, the intraductal (ID) type (ID‐FCSEMS) is placed within the bile duct, allowing stable placement at the stricture while avoiding side‐branch obstruction, and is removed after a fixed period to achieve stricture resolution [9]. Although several Western studies have reported favorable results for benign strictures, clear guidelines are lacking in Asia, where LDLT predominates. Moreover, optimal stent duration and strategies to prevent restenosis remain undefined, highlighting the need for further evaluation of efficacy, safety, and economic burden in clinical practice.

Against this background, we retrospectively evaluated ID‐FCSEMS for ABS after LDLT at our institution, focusing on clinical outcomes, safety, restenosis, and economic impact.

## 2 Materials and Methods

### 2.1 Patients

**FIGURE 1:**
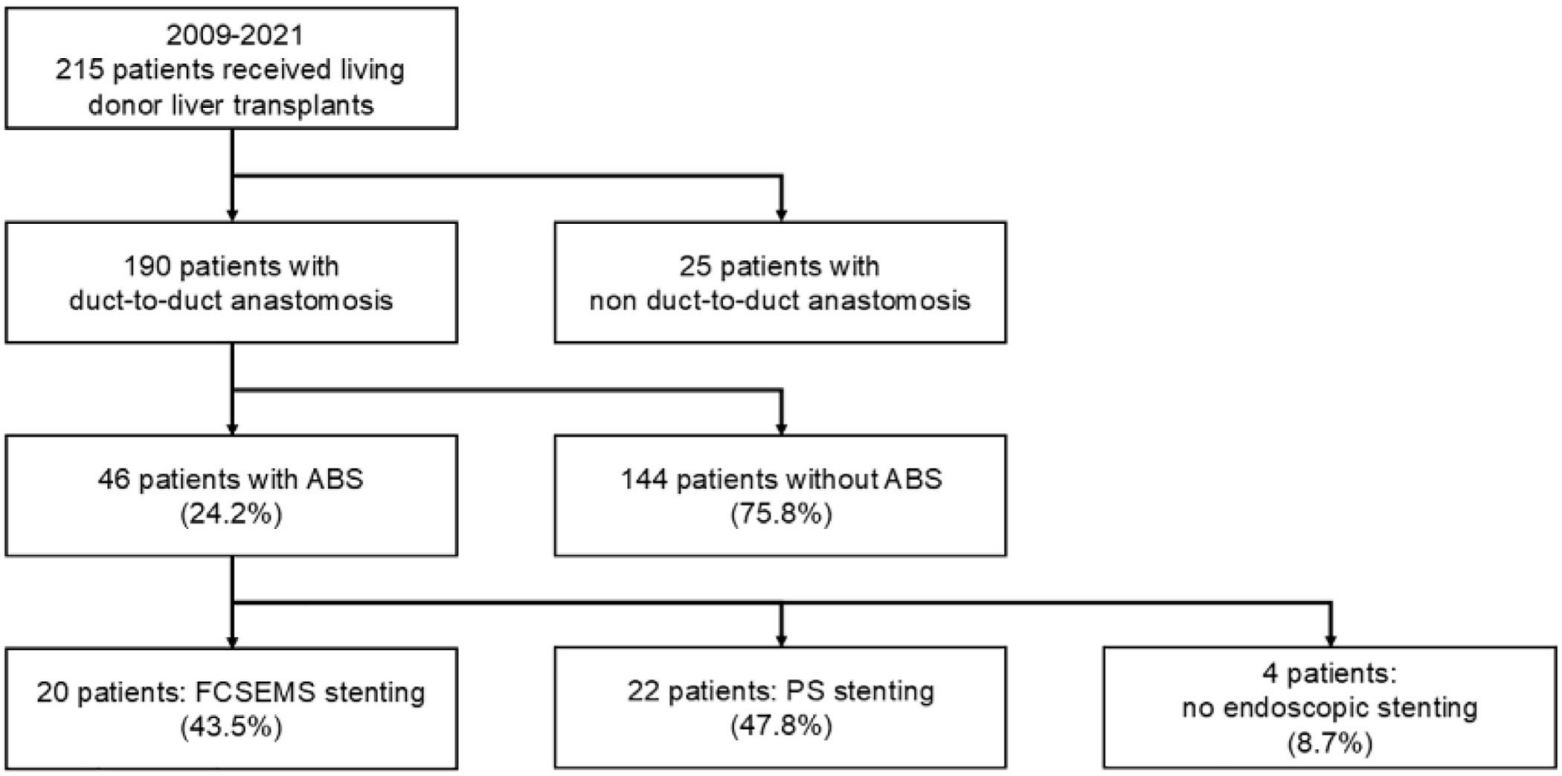
Flow diagram of patient selection. ABS, anastomotic biliary stricture; FCSEMS, fully covered self‐expandable metal stent; PS, plastic stent.

This single‐center, retrospective, observational study included adult patients who underwent LDLT and duct‐to‐duct biliary anastomosis at our institution between January 1, 2009, and December 31, 2021, and developed ABS (Figure 1). ABS was defined as cases in which patients developed abnormal laboratory findings during follow‐up (elevated bilirubin or hepatobiliary enzymes), jaundice, or clinical signs of cholangitis, and the physician performing cholangiography confirmed the presence of an anastomotic stricture.

The observation period was from January 1, 2009, to August 31, 2024. Data were collected from medical records and endoscopy reports.

### 2.2 Endoscopic Procedures

The choice of stent type (PS or FCSEMS) was made at the discretion of the attending endoscopist based on device availability and the institutional practice at the time of each procedure (including the characteristics of the stenosis and the clinical situation).

All patients received intravenous prophylactic antibiotics before undergoing endoscopic retrograde cholangiopancreatography (ERCP). ERCP was performed under moderate sedation with intravenous midazolam and pethidine hydrochloride using a side‐viewing duodenoscope (JF‐260 V, TJF‐260 V, and TJF‐Q290V; Olympus Medical Corporation, Tokyo, Japan). Following guidewire‐assisted biliary cannulation, the bile duct anastomotic stricture was dilated using a 6‐ or 8‐mm dilation balloon (REN; Kaneka Medics Co., Ltd., Osaka, Japan).

**FIGURE 2:**
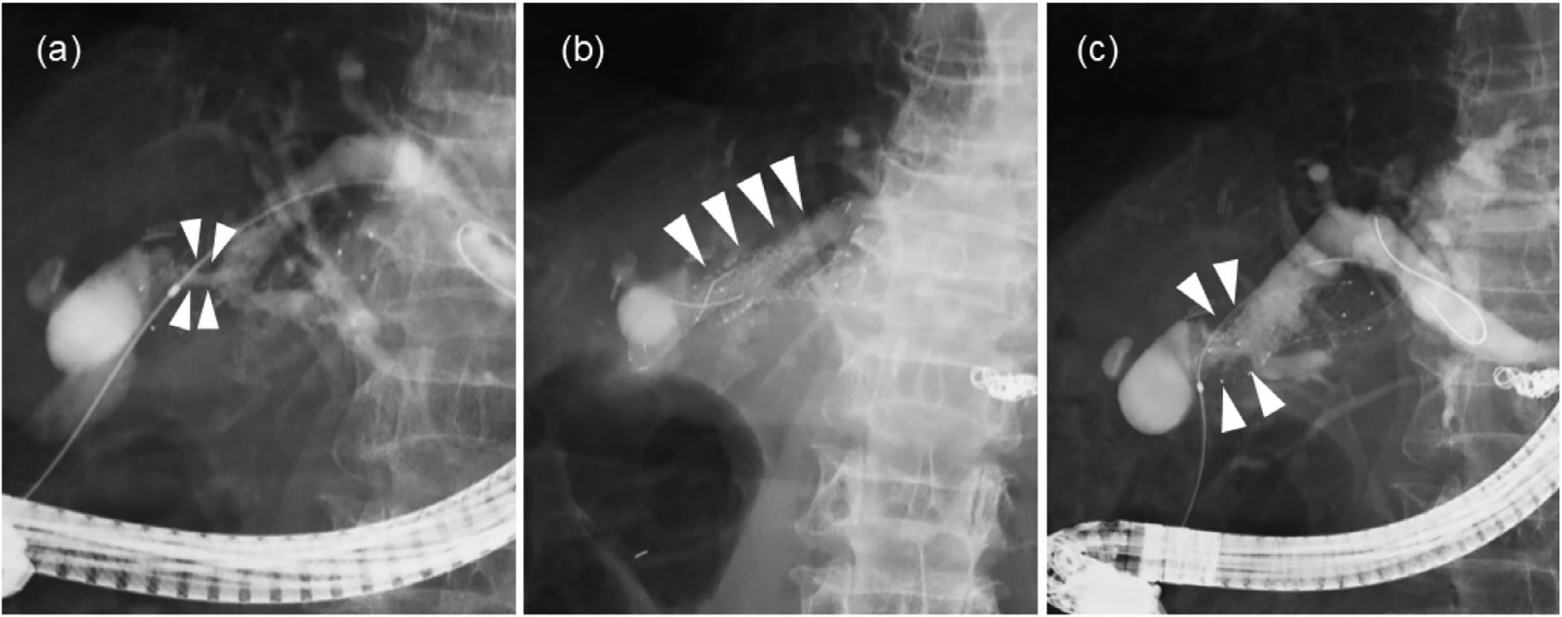
A 52‐year‐old woman with a bile duct anastomotic stricture after living donor liver transplantation with a left lobe graft. She had previously undergone plastic stenting with percutaneous transhepatic cholangiographic drainage rendezvous and 11 endoscopic retrograde cholangiopancreatography sessions over 7 years. (a) Cholangiogram before fully covered self‐expandable metal stent (FCSEMS) placement showing that the B2/B3 confluence and the anastomotic site were far apart, with no risk of side branch obstruction. A portal stent was also observed on the dorsal aspect of the bile duct. (b) FCSEMS placed in the left lateral segment of the bile duct. (c) Cholangiogram at FCSEMS removal after 16 weeks demonstrating improvement of the anastomotic stricture. The patient has remained free of recurrence for more than 53 months.

We selected the BONASTENT M‐intraductal (Standard Sci Tech Inc., Seoul, South Korea) as the ID‐FCSEMS. This ID‐FCSEMS is dumbbell‐shaped to prevent stent migration and side‐branch obstruction [10]. Diameters were 8, 10, or 12 mm, and the length was 30–70 mm in 10‐mm increments. The width and length of the FCSEMS were determined by the operator based on cholangiography results, and the FCSEMS was inserted via a guidewire. The central radiopaque marker was then aligned with the stenotic segment and left in place (Figure 2b).

The length of the FCSEMS was selected to avoid obstruction of the bile duct branches; however, if branch obstruction was anticipated, a plastic rescue stent was placed. The FCSEMS body was retained within the bile duct, and the lasso was retained in the duodenum via the papilla. Endoscopic sphincterotomy was not performed after LDLT in all cases.

The retention period for the FCSEMS was set at 16 weeks, in accordance with a previous study [11], and ERCP was scheduled for removal of the ID‐FCSEMS at 16 weeks. Cholangiography was performed, and the disappearance of the ABS was confirmed (Figure 2c). The dwell time of 16 weeks was based on previous studies, including [11], which suggested an optimal balance between adequate stricture remodeling and safe stent removal, and is consistent with reports recommending approximately 3–4 months of FCSEMS placement for benign biliary strictures.

Follow‐up was continued every 3–6 months after endoscopic treatment, and ERCP was performed if recurrence was suspected and biliary drainage was required.

The PS group comprised patients who underwent staged PS placement and met the diagnostic criteria for ABS. The number of ERCP procedures and stent replacements was determined at the discretion of the attending physician based on standard clinical practice.

### 2.3 Definition of Events

Technical success was defined as the successful placement of FCSEMS in the stenotic segment. Clinical success was defined as the absence of anastomotic stricture at the time of FCSEMS removal, confirmed by contrast imaging and determined by the physician performing the procedure.

Adverse events related to stent insertion were evaluated according to the guidelines of the American Society for Gastrointestinal Endoscopy [12]. Early adverse events occurred within 30 days of stent placement, and late events occurred thereafter.

Recurrence of stricture was defined as the presence of stricture confirmed by cholangiography after the initial clinical success.

As there are no established procedures for stricture resolution following PS placement, such cases were excluded from the analysis of stricture resolution rates.

Clinical outcomes—including stricture resolution, recurrence, number of ERCP sessions, total number of stents used, and adverse events during all ERCP procedures performed within the 1‐year observation period (including initial placement, removal, and reinsertion)—were compared between the FCSEMS and PS groups. For the 1‐year outcome analysis, patients with ≥12 months of follow‐up after stent removal were included.

For cost comparison, in the FCSEMS group, reimbursement points (Japanese medical fee schedule; 1 point = 10 yen), number of hospitalizations, and total inpatient days were calculated from initial stent placement to 2 years post‐placement, whereas in the PS group, these variables were calculated for any 2‐year period during follow‐up.

### 2.4 Statistical Analyses

Continuous variables are expressed as medians (ranges), and categorical variables as numbers (%). Group comparisons were performed using the Mann–Whitney U test for continuous variables and either the Fisher's exact test or Mann–Whitney U test for categorical variables, as appropriate. The open period after FCSEMS removal was determined using the Kaplan–Meier method. All analyses were performed using the JMP 17 software (JMP Statistical Discovery LLC, Cary, NC, USA).

## 3 Results

Of the 215 LDLTs performed at our institution between January 2009 and December 2021, duct‐to‐duct biliary anastomosis was performed in 190 patients. Among these 46 (24%) developed ABS (Figure 1). Of the 46 patients with ABS, 20 underwent FCSEMS placement, and 22 underwent PS placement, whereas endoscopic stent placement was not feasible in 4 patients. Baseline characteristics of the overall ABS cohort and FCSEMS and PS groups are summarized in Table 1. Briefly, the median age of patients with ABS was 58 (range, 38–70) years, 59% were male, and the median time to ABS onset was 4 (range, 0–42) months. Based on bile duct diameter and stricture length, the attending endoscopist selected FCSEMS 8–10 mm in diameter (median 10 mm) and 40–60 mm in length.

**TABLE 1:** Characteristics of 46 patients.

	All *n* = 46	FCSEMS *n* = 20	PS *n* = 22	*p*‐Value
Age, median (range), years	58 (38–70)	60.5 (43–66)	58 (38–70)	0.787
Sex, *n* (%)				0.673
Male	27 (58.7)	13 (65.0)	12 (54.5)	
Female	19 (41.3)	7 (35.0)	10 (45.5)	
Primary disease, *n* (%)				0.480
Hepatitis B	7 (15.2)	4 (22.2)	3 (13.6)	
Hepatitis C	16 (34.8)	7 (38.9)	8 (36.4)	
Alcohol‐associated liver disease	8 (19.6)	4 (22.2)	4 (18.2)	
Metabolically‐dysfunction‐associated steatotic liver disease	6 (10.9)	2 (11.1)	3 (13.6)	
Primary biliary cholangitis	5 (10.9)	3 (16.7)	2 (9.1)	
Others	4 (8.6)	0	2 (9.1)	
Presence of HCC, *n* (%)	16 (34.8)	8 (40.0)	6 (27.3)	0.581
Liver graft, *n* (%)				0.009
Right lobe	20 (43.5)	4 (16.7)	13 (59.1)	
Left lobe	26 (56.5)	16 (83.3)	9 (40.9)	
Time to ABS onset (months), median (range)	4 (0–42)	7 (1–42)	3.5 (1–15)	0.248

Of the 46 patients diagnosed with ABS, 20 underwent FCSEMS placement, and 22 underwent PS placement. Endoscopic stent placement was not feasible in four patients. FCSEMS, fully covered self‐expandable metal stent; PS, plastic stent; HCC, hepatocellular carcinoma; ABS, anastomotic biliary stricture.

Treatment outcomes in the FCSEMS group are presented in Table 2. FCSEMS placement was technically successful in all 20 patients. Early adverse events consisted of cholangitis in five patients, all of which resolved with conservative treatment, including antibiotic administration. No cases of post‐ERCP pancreatitis (PEP) occurred in patients who underwent FCSEMS placement. In the PS group, three patients developed mild PEP during repeated ERCP sessions; all recovered with conservative treatment. There was no standardized prophylactic protocol for PEP prevention; rectal NSAIDs were used in one case at the discretion of the attending endoscopist. Late adverse events in the FCSEMS group included distal stent migration in one patient and obstructive cholangitis in one patient. The latter underwent FCSEMS removal 6 weeks after placement. FCSEMS removal was performed in 17 patients, including two with early removal and one who died of other causes before the scheduled removal; stricture resolution was achieved in 16 of these 17 patients.

**TABLE 2:** Summary of treatment outcomes with fully covered self‐expandable metal stent (FCSEMS).

		FCSEMS
Technical success, *n* (%)		20 (100)
Early adverse events, *n* (%)		
Cholangitis		5 (25)
Late adverse events, *n* (%)		
Stent migration		1 (5)
Obstructive cholangitis		1 (5)
Stricture resolution rate, *n* (%)		16 (94.1)
Duration of stenting (weeks), median (range)		16 (6–16)
Post‐stricture follow‐up period (months), median (range)		52 (0–79)
Recurrence of stricture, *n* (%)		2 (12.5)
Duration until recurrence (days), median (range)		157 (13–288)

FCSEMS was placed in 20 patients and removed in 17 patients; stricture resolution was achieved in 16 of these 17 patients. Adverse events in this table represent those occurring at the time of the initial FCSEMS placement. Additional adverse events observed during subsequent procedures (e.g., FCSEMS reinsertion) are included in the 1‐year outcome analysis shown in Table 3. FCSEMS, fully covered self‐expandable metal stent.

**FIGURE 3:**
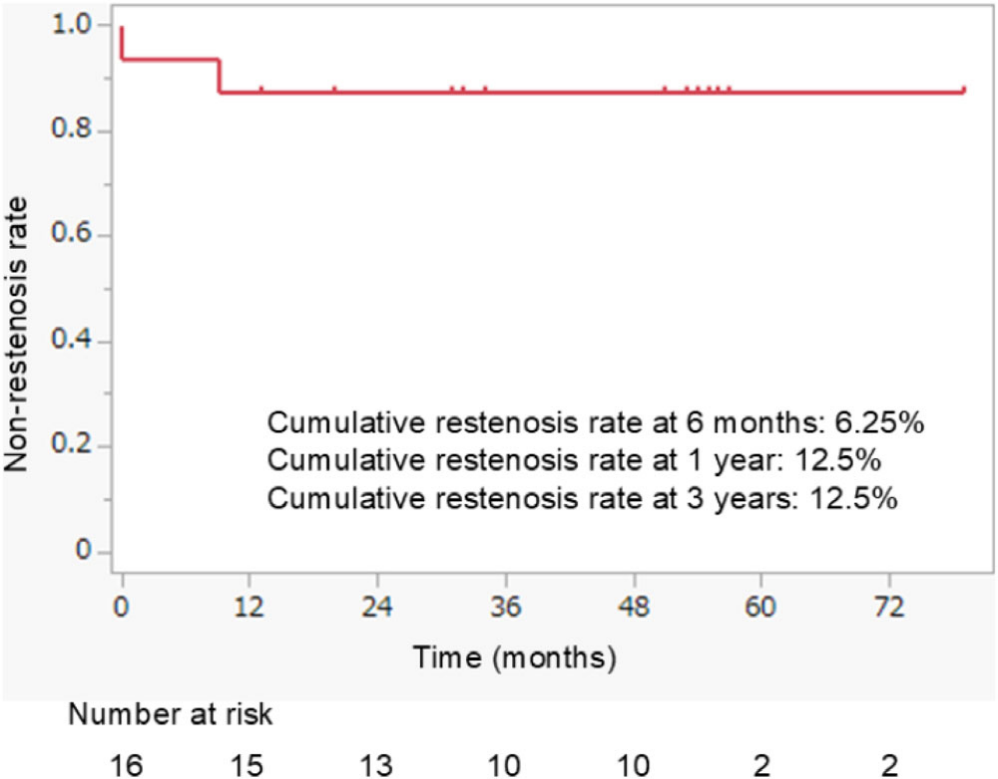
Cumulative restenosis rate after fully covered self‐expandable metal stent (FCSEMS) removal in patients with clinical success. The cumulative restenosis rates were 6.25% at 6 months and 12.5% at 1 and 3 years after FCSEMS removal.

The median follow‐up period after FCSEMS removal was 52 (range, 0–79) months, with two cases showing restenosis. The median time to restenosis was 157 (range, 13–288) days. Of these two cases, one underwent FCSEMS reinsertion and achieved favorable long‐term patency, whereas the other was treated with PS placement and died 14 months later from unrelated causes. The cumulative restenosis rate at 6 months after FCSEMS removal was 6.25%, and the cumulative restenosis rates at 1 and 3 years were 12.5%, respectively (Figure 3).

A comparison of 1‐year clinical outcomes between the FCSEMS and PS groups, including stricture resolution rate, recurrence, number of ERCP sessions, and adverse events, is summarized in Table 3.

**TABLE 3:** Comparison of 1‐year clinical outcomes between the fully covered self‐expandable metal stent (FCSEMS) and plastic stent (PS) groups.

		FCSEMS *n* = 17^a^	Plastic Stent *n* = 15^a^	*p*‐Value
Stricture resolution within 1 year, *n* (%)		17 (100)	0 (0)	<0.001
Recurrence within 1 year, *n* (%)		2 (11.8)	N/A^b^	
Median no. of ERCP sessions per patient (range)		2 (2–4)	2 (1–5)	0.488
Median no. of stents used per patient (range)		2 (1–4)	2 (1–7)	0.058
Median duration of stent placement (weeks)		16 (7–32)	26 (1–65)	0.208
Any adverse event, *n* (%)		10 (58.8)	13 (86.7)	0.122
Early adverse events, *n* (%)^§^		8 (47.1)	5 (33.3)	0.491
Cholangitis		8 (47.1)	3 (20.0)	0.148
PEP		0 (0)	2 (13.3)	0.212
Late adverse events, *n* (%)^§^		3 (17.7)	12 (80.0)	0.001
Stent migration		1 (5.9)	4 (26.7)	0.161
Obstructive cholangitis		2 (11.8)	11 (73.3)	<0.001

^a^ Patients who were followed up for at least 12 months after stent removal were included in the 1‐year outcome analysis. Cases with less than 12 months of follow‐up were not evaluated.

^b^ Restenosis was evaluated only in patients who achieved initial stricture resolution. Because no patients in the PS group achieved stricture resolution, the restenosis rate was not applicable (N/A).

^c^ Adverse events during all ERCP procedures within the 1‐year observation period—including initial stent placement, removal, and reinsertion—were evaluated on a per‐patient basis; early events were defined as those within 30 days, and late events as those occurring thereafter.

Percentages were calculated using the total number of patients in each group as the denominator, except for restenosis, which was calculated among patients who achieved stricture resolution. FCSEMS, fully covered self‐expandable metal stent; PS, plastic stent; ERCP, endoscopic retrograde cholangiopancreatography; PEP, post‐ERCP pancreatitis.

Table 4 shows a comparison of ABS‐related medical costs between the FCSEMS and PS groups. The total number of medical reimbursement points, number of hospitalizations, and total inpatient days were compared between 15 patients in the FCSEMS group who were followed for more than 2 years after FCSEMS removal and 15 patients in the PS group who were followed for more than 2 years during any 2‐year period of follow‐up. The median number of reimbursement points was 305,084 in the FCSEMS group (median, two hospitalizations and 27 total inpatient days) and 367,321 in the PS group (median, three hospitalizations and 24 total inpatient days). There were no significant differences between the FCSEMS and PS groups in terms of reimbursement points (*p* = 0.2902), number of hospitalizations (*p* = 0.2643), and total inpatient days (*p* = 0.3504).

**TABLE 4:** Comparison of fully covered self‐expandable metal stent (FCSEMS) and plastic stent (PS) costs.

	FCSEMS *n* = 15	PS *n* = 15	*p*‐Value
Total reimbursement points, median (range)	305,084 (119,701–639,906)	367,321 (127,078–3,258,673)	0.290
Number of hospitalizations, median (range)	2 (1–4)	3 (1–9)	0.264
Total inpatient days, median (range)	27 (5–60)	24 (9–225)	0.350

Total reimbursement points (defined by the Japanese medical fee schedule; 1 point = 10 yen), number of hospitalizations, and total inpatient days were compared between the FCSEMS group for 2 years after FCSEMS implantation and the PS group for any 2‐year period during follow‐up. FCSEMS, fully covered self‐expandable metal stent; PS, plastic stent.

## 4 Discussion

In this study, ID‐FCSEMS for ABS after LDLT achieved high technical and clinical success rates and a low cumulative restenosis rate during long‐term follow‐up. The cumulative restenosis rates after FCSEMS removal were 6.25% at 6 months and 12.5% at 1 and 3 years, suggesting durable stricture resolution in most patients.

Traditionally, stepwise dilation using PSs has been the standard treatment for ABS [4, 5, 6], but the prolonged treatment duration and frequent procedures required have been problematic [13]. FCSEMS are expected to reduce the number of treatments, achieve early restenosis relief, and improve patient quality of life [7, 9, 13, 14, 15, 16].

The stricture resolution rate for PSs reported in previous studies ranges from 63% to 100%, whereas that for FCSEMS ranges from 53% to 100%. Additionally, the restenosis rate for PSs ranges from 0% to 37.5%, whereas that for FCSEMS ranges from 0% to 37.5% [7, 9, 13, 15, 16, 17, 18], indicating that FCSEMS is not necessarily superior. However, recent reports indicate that outcomes of FCSEMS improve with appropriate selection and longer dwell times. Lim et al. [18] reported that in patients with ID‐type FCSEMS for bile duct anastomotic strictures after LDLT, the restenosis rate was 21.4% in those with a stent placement duration of longer than 120 days, which was significantly lower than the 71.4% observed in patients with a duration of 120 days or less (*p* = 0.03). Matsumoto et al. [19] reported that ID FCSEMS placed for 4 months in patients with benign bile duct strictures resulted in a low restenosis rate of 16%, demonstrating efficacy in maintaining patency over the medium to long term. In contrast, Sissingh et al. [17] reported that restenosis after FCSEMS placement was 33% at a median of 133 days, comparable to that of PSs, suggesting that variations in placement duration and patient characteristics may influence outcomes. Based on these results, FCSEMS treatment for ABS is advantageous in suppressing restenosis when left for 120 days (approximately 4 months) or longer, without compromising safety [8]. In our study, however, the stents remained in place for only 16 weeks, yet stricture resolution was achieved in most cases.

The complications associated with FCSEMS placement include pancreatitis, obstructive cholangitis due to side branch obstruction, and stent migration. Advantages of ID‐type FCSEMS include reduced risk of pancreatitis, avoidance of side branch obstruction, stent stability, and ease of removal [9, 17]. In cases where a non‐ID‐type FCSEMS was used for postoperative benign bile duct strictures, complications included cholangitis in 33%, pancreatitis in 6%, and a stent dislodgement rate of approximately 17% [20]. In the placement of ID‐type FCSEMS, the incidence of pancreatitis as an adverse event was low [9, 21, 22], and cholangitis occurred in 3%–4% of cases; however, all cases improved with conservative treatment [18, 23]. ID‐type FCSEMS, which does not pass through the papilla, significantly contributed to the prevention of pancreatitis as a complication. Stent migration was observed at a relatively high rate of 2.8%–46.7% [8, 16, 18, 22, 23, 24]. ABS is typically short with localized strictures, leading to inadequate fixation and subsequent migration of the stent, which not only fails to achieve adequate dilation but may also induce obstructive cholangitis. The ID‐FCSEMS used in this study was designed to reduce the risk of migration and bile duct branch obstruction compared with conventional stents. In this study, late complications, such as distal displacement during placement or obstructive cholangitis, were rare and manageable with appropriate intervention; in particular, the placement of a plastic rescue stent in the side branch for rescue purposes in some cases was considered effective in preventing the onset of obstructive cholangitis.

In the 1‐year comparison between the FCSEMS and PS groups, the FCSEMS group showed superior stricture resolution and fewer late adverse events, while early adverse events and procedural frequency were similar between the two groups.

From a healthcare economic perspective, there were no statistically significant differences in the reimbursement points, number of hospitalizations, or total inpatient days between the FCSEMS and PS groups. However, the median number of hospitalizations was lower in the FCSEMS group compared with the PS group, suggesting potential savings in medical resources owing to reduced procedural requirements. This finding is consistent with the results of previous studies reporting that FCSEMS may help reduce overall healthcare costs [13, 25, 26]. Going forward, it will be necessary to conduct multicenter prospective studies that focus not only on clinical outcomes but also on healthcare economics and quality of life. The development of a stent selection algorithm tailored to factors such as the length and location of the stenotic segment and the type of graft is expected to contribute to further improvements in treatment outcomes. Additionally, improvements in stent design may help reduce complications, such as cholangitis and stent migration. By incorporating these technical advancements into clinical practice, treatment strategies for ABS after LDLT are anticipated to be further refined.

The limitations of this study include its single‐center, retrospective, observational design, limited number of cases, and the fact that stent selection was based on the discretion of the treating physician, which may introduce bias. Future prospective studies, including randomized controlled trials, are required to clarify the optimal duration of use and the selection criteria for FCSEMS.

## 5 Conclusion

In this retrospective study, we evaluated the efficacy and safety of ID‐FCSEMS for ABS after LDLT. ID‐FCSEMS appears to be an effective and safe treatment option, particularly for rapid stricture relief. Large prospective multicenter studies should establish a clinical treatment algorithm for ABS, including placement duration and restenosis prevention.

## Author Contributions

**Conceptualization**: Akane Shimakura, Eisuke Ozawa, Kosuke Takahashi, and Hisamitsu Miyaaki; **methodology**: Akane Shimakura, Eisuke Ozawa, and Kosuke Takahashi; **software**: Akane Shimakura; **validation**: Akane Shimakura, Eisuke Ozawa, Kosuke Takahashi, Yasuhiko Nakao, Masanori Fukushima, Ryu Sasaki, Masafumi Haraguchi, Satoshi Miuma, and Hisamitsu Miyaaki; **formal analysis**: Akane Shimakura, Eisuke Ozawa, and Kosuke Takahashi; **investigation**: Akane Shimakura, Mizuki Kitagawa, Kosuke Takahashi, Yasuhiko Nakao, Masanori Fukushima, Ryu Sasaki, Masafumi Haraguchi, Akihiko Soyama, and Susumu Eguchi; **resources**: Akane Shimakura, Mizuki Kitagawa, Kosuke Takahashi, Yasuhiko Nakao, Masanori Fukushima, Ryu Sasaki, Masafumi Haraguchi, Akihiko Soyama, and Susumu Eguchi; **data curation**: Akane Shimakura; **writing – original draft preparation**: Akane Shimakura; **writing – review and editing**: Akane Shimakura and Eisuke Ozawa; **visualization**: Akane Shimakura; **supervision**: Eisuke Ozawa, Satoshi Miuma, Akihiko Soyama, Susumu Eguchi, and Hisamitsu Miyaaki; **project administration**: Akane Shimakura and Eisuke Ozawa; **funding acquisition**: none. All the authors have read and agreed to the published version of the manuscript.

## Conflicts of Interest

The authors declare no conflicts of interest.

## Funding

The authors received no specific funding for this work

## Ethics Statement

The study protocol was approved by the Clinical Research Ethics Committee of Nagasaki University Hospital on November 29, 2022 (approval no. 22112118).

## Consent

Informed consent for bile duct stent placement was obtained from all patients. This study was conducted in accordance with the principles of the Declaration of Helsinki.

## Clinical Trial Registration

N/A.
